# Apexification of an Immature Permanent Incisor with the Use of Calcium Hydroxide: 16-Year Follow-Up of a Case

**DOI:** 10.1155/2015/984590

**Published:** 2015-06-11

**Authors:** Camila Maggi Maia Silveira, Cátia Cilene Nass Sebrão, Larissa Soares Reis Vilanova, Alfonso Sánchez-Ayala

**Affiliations:** ^1^Dental School, Center of Higher Education of Campos Gerais, Avenida General Carlos Cavalcanti, No. 8000, Ponta Grossa, PR, Brazil; ^2^Department of Oral Health, Federal University of Goiás, Primeira Avenida, s/n, Goiânia, GO, Brazil; ^3^Department of Dentistry, State University of Ponta Grossa, Avenida General Carlos Cavalcanti, No. 4748, Ponta Grossa, PR, Brazil

## Abstract

Apexification is a process of forming a mineralized apical barrier and had been performed by using calcium hydroxide paste, due to its biological and healing performances in cases of existent trauma. This clinical report aims to report the results of a 16-year follow-up study of an apexification treatment applied to nonvital tooth 22 of a healthy 8-year-old male after a trauma. Clinical inspection of the tooth showed fractures of the incisal edge and mesial angle, absence of coronal mobility, and negative pulp vitality under cold testing. Radiographic analysis of the root revealed incomplete apex formation. The possibility of fracture into the root or luxation injury was rejected, and the diagnosis of pulp necrosis was verified. Apexification by calcium hydroxide and subsequent endodontic treatment were planned. Initial formation of the mineralized apical barrier was observed after 3 months, and the barrier was considered to be completed after 8 months. Clinical, radiographic, and CBCT examinations after 16 years verified the success of the treatment, although the choice of calcium hydroxide for apexification treatment is discussed.

## 1. Introduction

Trauma to the anterior teeth is a relatively common occurrence during childhood. Depending on its magnitude, it may produce concussion, luxation, fracture, or avulsion of the teeth, leading, in more severe cases, to necrosis of the pulp tissue [1]. When pulp regeneration or repair is not possible, the endodontic treatment of immature permanent necrotic teeth is more time-consuming and technically more difficult than conventional procedures because these teeth present widened root canals and open apices [1]. The tooth roots may also suffer external infection-related (inflammatory) root resorption or alterations of their formation during treatment [2].

In cases of infected pulps, it is necessary to use a specific dressing material to neutralize the bacteria and their products and to stimulate the apexification process by forming a mineralized apical barrier so that the subsequent condensation of gutta-percha can be properly achieved [3]. Traditionally, apexification had been performed by using calcium hydroxide paste, due to its biological and healing performances [4]. Regardless of the proprietary brand, calcium hydroxide has been successfully used for apical barrier formation in 74–100% of cases [5]. Additionally, 86% of the treated teeth survived after a follow-up of 5 years.

However, the suitability of calcium hydroxide paste use for apexification has been questioned because it involves a long treatment time and the prognosis is always uncertain. The average length of time for apical barrier formation ranges from ~3 to 17 months, necessitating multiple visits for material replacement and delays in the construction of the definitive restoration [5]. Long-term exposure of the tissue to calcium hydroxide may weaken the root structure, resulting in cervical fractures, as well as inducing periapical bone necrosis when there is overfilling of the material [6].

Nevertheless, the authors believe that calcium hydroxide paste, if properly used, may still be a suitable material for apexification. Currently, the use of other alternative materials, such as mineral trioxide aggregate (MTA) or calcium hydroxide microspheres, may still be restricted due to socioeconomic or regional conditions. Therefore, the aim of this study was to report the 16-year follow-up data of an apexification treatment applied to a permanent incisor of a young patient treated with calcium hydroxide.

## 2. Case Report

In March 1998, a healthy 8-year-old boy fell on a concrete soccer field while playing at school, fracturing his left lateral incisor through the frontal impact of his maxilla on the ground. The patient reportedly suffered a small cut in the superior labial mucosa, slight bleeding at the gingival sulcus, and local pain and numbness of the anterior maxillary teeth. At an emergency dentistry service, the fractured tooth was provisionally restored by covering the exposed dentin with resin-modified glass-ionomer cement. The parents were recommended to monitor the pulpal conditions of all involved teeth, and the pain almost disappeared after 3 days. However, 10 days later, spontaneous tooth pain of the central and lateral maxillary incisors at night led the patient's parents to seek endodontic care for the child.

**Figure 1:**
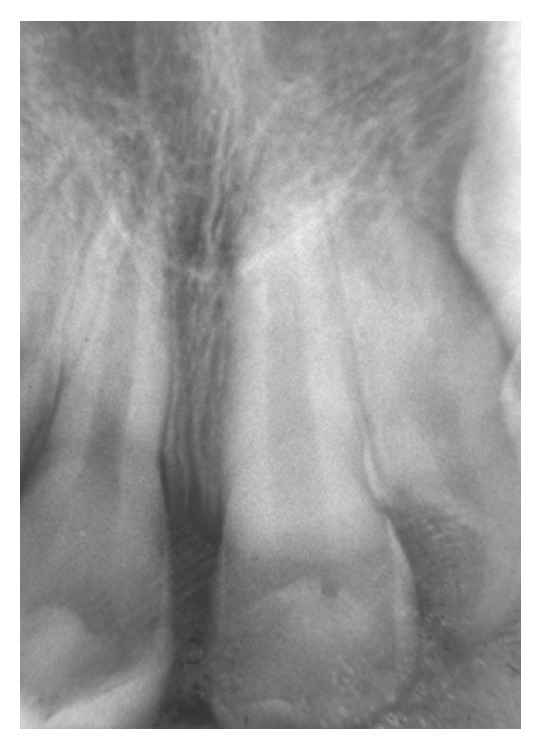
The initial periapical radiograph revealed the absence of fracture lines on the root structures, increased periodontal ligament spaces apically, or any radiolucent lesion in apical area of the injured incisors (first session; March 1998).

Clinical inspection verified the fracture of incisal edge and mesial angle of tooth 22, with loss of structure at the enamel and dentin but without pulpal exposure. According to Miller's scale (within 0–3), the dental mobility scores of teeth 12 and 22 were rated as 1-2. These teeth did not respond to cold vitality testing using frozen cotton pellets Endo-Frost (Coltène/Whaledent, Langenau, Banden-Württemberg, Germany), but slight tenderness to vertical percussion was recorded. Radiographic analysis (Eastman Kodak Company, Rochester, NY, USA) revealed no fracture lines on the root structures or increased periodontal ligament spaces apically; thus, the possibility of a luxation injury was rejected (Figure 1). The other anterior teeth were asymptomatic. A root canal treatment was chosen for tooth 12. Calcium hydroxide apexification was planned for tooth 22, due to the presence of a widened root canal and an immature open apex (Figure 1). The written consent was provided by the parents after the procedures were explained.

**Figure 2:**
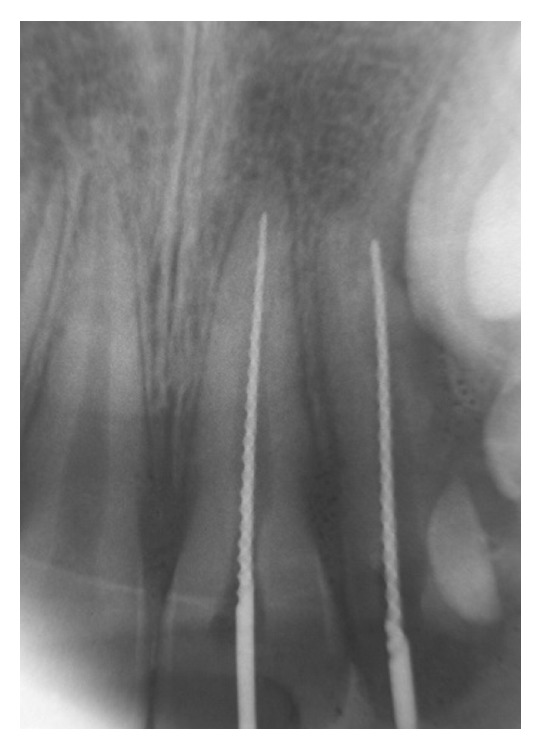
Periapical radiograph of the initial exploration of root canals (first session; March 1998).

In a first session, absolute isolation of the operative field was achieved by using a rubber dam and clamps held on the premolars teeth. Coronal pulp chamber accesses were made with a diamond bur 1014# (KG Sorensen, São Paulo, SP, Brazil) in a high-speed hand piece irrigated with an air-water spray. Despite the vitality signals previously described, the access was initially made without anesthesia to confirm the pulp condition* in situ*. During the procedures, the patient related a mild sensitivity. Afterwards, mepivacaine HCl 3% (Mepivalem, Dentsply Pharmaceutical, Catanduva, SP, Brazil) was employed, but without vasoconstrictor to avoid a probable transient ischemia. However, the pulp tissue was found to be ischemic and without bleeding, confirming the diagnosis of pulp necrosis in both teeth (Figure 2).

**Figure 3:**
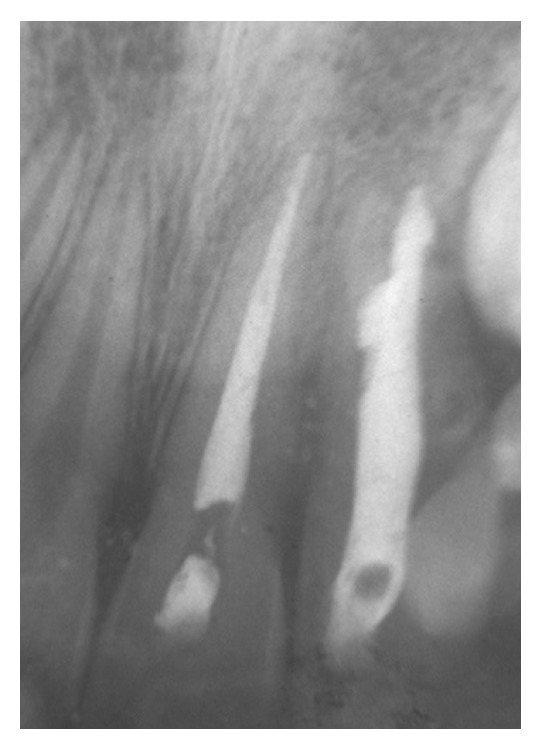
Periapical radiograph showing the root canal treatment of tooth 21 after 1 month (third session; April 1998).

Root canal therapy to tooth 12 was conventionally accomplished in two visits. Canal patency was achieved with a Senseus FlexoFile Endodontic Instrument 10# (Dentsply/Maillefer, Johnson City, TN, USA). The working length was established 0.5 mm short of apex, and instrumentation was performed with a manual step-back technique. The canal was alternately irrigated with 1% sodium hypochlorite (AFER, Ponta Grossa, PR, Brazil) and 0.9% sodium chloride (Segmenta Farmacêutica, Ribeirão Preto, SP, Brazil). The apical preparation was performed up to 40# by using a K-type file (Dentsply/Maillefer), and the canal was thoroughly dried with multiple paper points. In a second session, the canal was obturated with a zinc-oxide eugenol-based sealer Endoseal (Ultradent Products, Inc., South Jordan, UT, USA) and gutta-percha cones (Dentsply/Maillefer), using the cold lateral obturation technique (Figure 3).

In the first visit, apexification of tooth 22 was simultaneously performed according to guidelines of the International Association of Dental Traumatology [7]. The pulp chamber and canal were irrigated with 1.0% sodium hypochlorite (AFER) to neutralize a probable septic content due to dentin exposition during fracture. A Senseus FlexoFile Endodontic Instrument 25# L25 mm (Dentsply/Maillefer) was used to confirm the absence of fissures or fracture into the root. Then, the odontometry was radiographically determined. Instrumentation of the canal was executed by removing the necrotic remaining pulp tissue, shaping the canal walls, cleaning under constant irrigation with 1.0% sodium hypochlorite (AFER) and 0.9% sodium chloride (Segmenta Farmacêutica), and drying with sterile absorbent paper points (Dentsply/Herpo, Petrópolis, RJ, Brazil).

A paste of calcium hydroxide manipulated at an adequate density with propylene glycol vehicle and iodoform (Biodinâmica Química e Farmacêutica, Ibiporã, PR, Brazil) was placed into the canal. The condensation and filling of material were completed with the aid of a small spatula and lentulo carrier 25# L25 mm (Dentsply/Maillefer). Appropriate filling was radiographically confirmed. Figure 3 shows the slight extrusion of the material into the periapical tissues. The tooth was temporarily sealed with zinc-oxide eugenol-base cement TempBond (Kerr Corp, Orange, CA, USA). After 24 h, the patient did not complain of pain or discomfort. The calcium hydroxide dressings were replaced after 1, 3, 6, and 8 months. The canal entry was covered with resin-modified glass-ionomer cement (GC Corporation, Tokyo, Japan).

**Figure 4:**
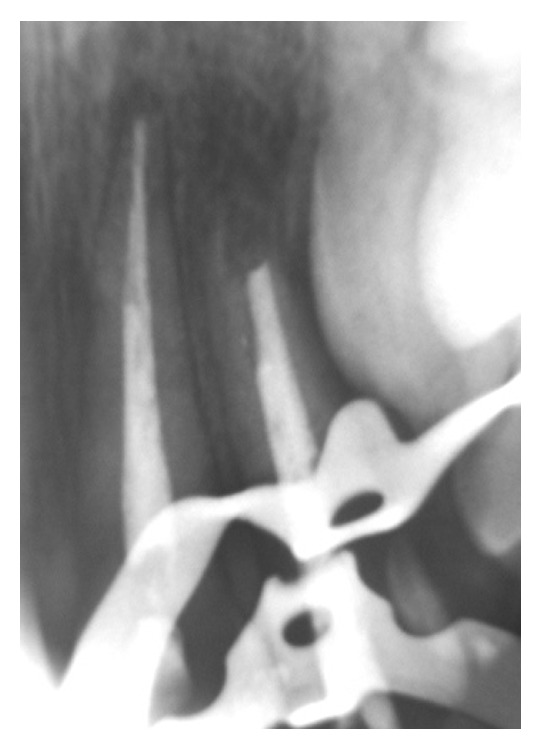
Periapical radiograph after 1 year of starting treatment showing the apical barrier formation after 8 months (seventh session; March 1999).

**Figure 5:**
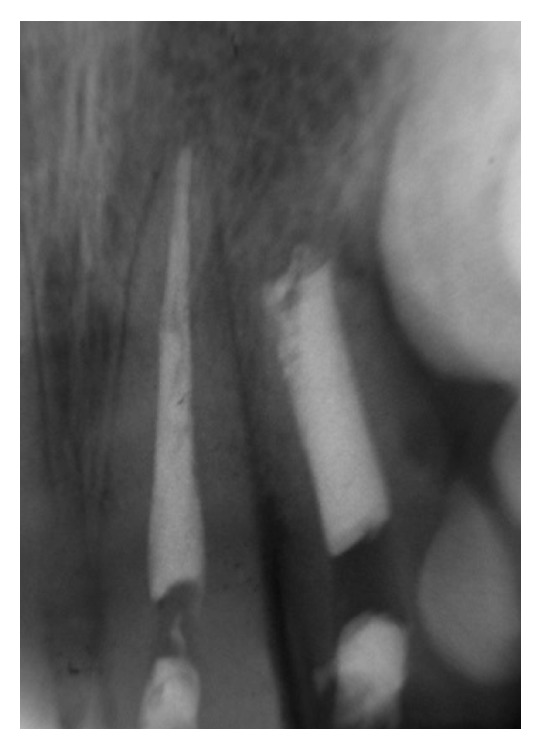
Periapical radiograph: root canal treatment finished in tooth 22 and tooth 21 in good condition after 1 year (seventh session; March 1999).

**Figure 6:**
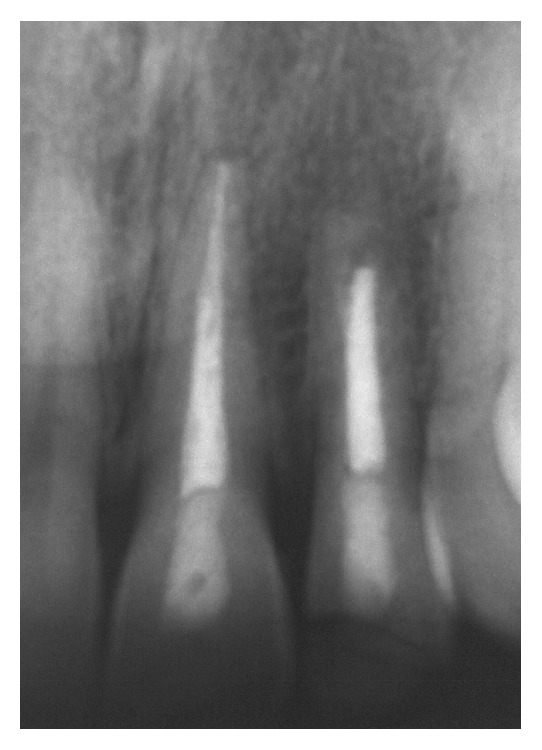
Radiograph of thirteen-year follow-up (March 2012).

**Figure 7:**
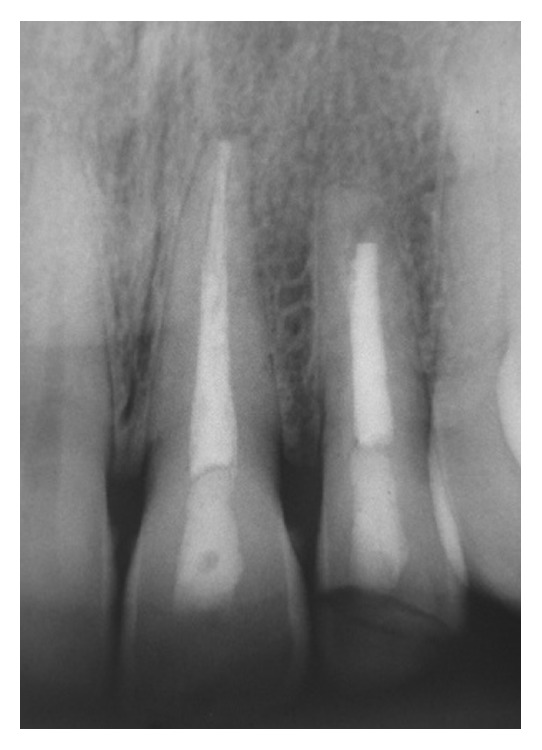
Radiograph showing the long-term success of apexification and root canal treatment of tooth 22 after fourteen and half years of follow-up and root canal treatment of tooth 21 after fifteen and half years (October 2013).

**Figure 8:**
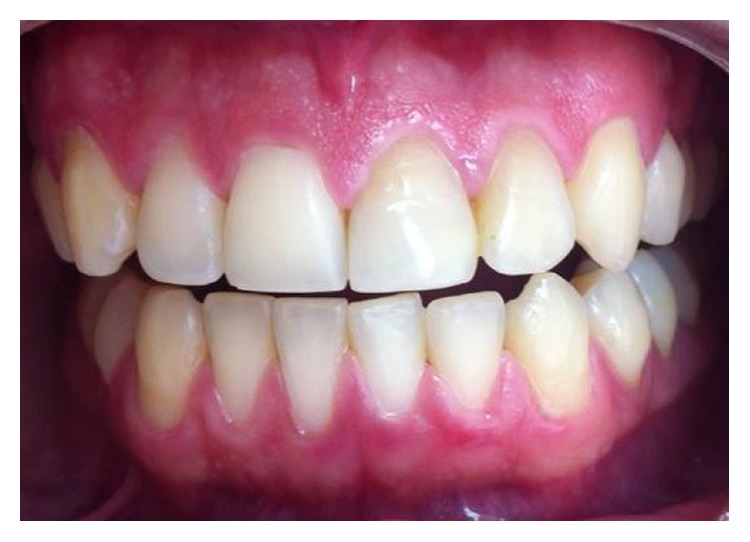
Clinical aspect showing the normal function of teeth 21 and 22 (October 2013).

**Figure 9:**
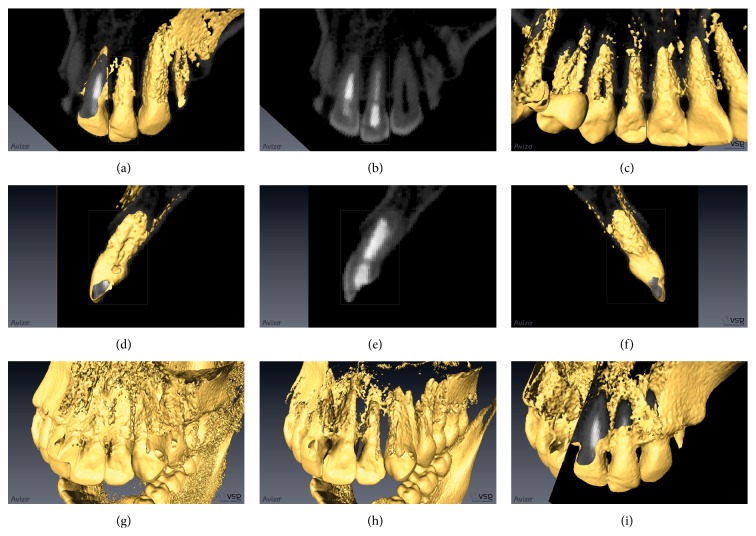
Cone beam computerized tomography and 3D rendering of tooth 22, visualized by the use of Avizo Fire software for Windows (Visualization Science Group, 33700 Mérignac, France). (a) 3D anterior view showing the apexification of tooth 22. (b) Anteroposterior slide in the same position of (a). (c) 3D posterior view. (d) 3D distal view. (e) Mesiodistal slide in the same position of (d). (f) Mesial view. (g) Frontal slide in the middle of tooth 22 showing a complete formation of periapical bone around apex. (h) The same view from (g), but with lower bone density performed by the software. It can be observed that the periapical bone was maintained. (i) Diagonal slides mixing 2D and 3D views, where the complete apexification and formation of periapical bone can also be found.

At 1 month, the initial restoration was removed and a new one was constructed with resin-based composite Z100 (3M-ESPE, Saint Paul, MN, USA). Within 3 months, the initial formation of mineralized apical barrier was radiographically observed. Exposure to calcium hydroxide was ended after 8 months, when the complete formation of a barrier was observed. After 4 months, the endodontic treatment was finished (Figures 4 and 5), and the coronal pulp chamber access was properly restored by using a resin-based composite Z100 (3M-ESPE). The patient did not come for controls and only returned to the office to change his aged restoration. Then, the long-term outcome was verified clinically and radiographically 13 years (Figure 6) and 14 and half years (Figures 7 and 8) after treatment. Moreover, the success of treatment was proved by means of a cone beam computerized tomography (CBCT) performed after 16 years and two months of the end of treatment.  Figure 9 shows the complete apexification and formation of periapical bone.

## 3. Discussion

The purpose of this paper was to show the capacity of calcium hydroxide to ensure the long-term success of apexification in a case study. In powder form, calcium hydroxide (molecular weight = 74.08) is a strong base (pH = 12.5–12.8) that has poor water solubility (≈ 1.2 gL^−1^ at 25°C) with thixotropic behavior and is insoluble in alcohol. It dissociates (dissociation coefficient = 0.17) into calcium (54.11%) and hydroxyl (45.89%) ions [3]. It was introduced as a biocompatible endodontic agent for direct pulp-capping in 1920 [3]. Since 1966, it has also been employed in apexification [8].

Apexification is not a static process, and the involved area undergoes years of rearrangement involving the apical bone, root tissues, and root-filling material [9]. However, there is limited evidence involving cases with long-term survival. A search of the PUBMED electronic database using the keywords “apexification” and “calcium hydroxide” without language limitation through November 2013 identified 209 papers, only a few of which reported a survival time of 5 years [10, 11], 8 years [12], 12 years [13], or 13 years [9].

Apexification requires the formation and maintenance of an apical calcified barrier, which consists of osteocementum or other bone-like tissue [3]. Under ideal conditions, residual pulp tissue and the odontoblastic layer may form a matrix, such that the subsequent calcification can be guided by the reactivated epithelial cell rests of Malassez [14] or nonperiapical pluripotent cells within bone [15]. Barrier formation also depends on the degree of inflammation and pulp necrosis, displacement at the time of trauma, and number of calcium hydroxide dressings, which can complicate (or at least delay) treatment [4].

Calcium hydroxide can induce healing conditions because of its antibacterial behavior. As a result of its high pH, the highly reactive hydroxyl ions produce damage to the bacterial cytoplasmic membrane by denaturing protein and destroying lipoproteins, phospholipids, and unsaturated fatty acids. Consequently, these actions lead to bacterial vulnerability and alteration of the nutrient transport and DNA [3]. Calcium hydroxide also hydrolyzes the toxic lipid A of bacterial endotoxin into atoxic fatty acids and amino sugars, thereby inactivating the inflammatory reaction and periapical bone resorption [16].

An alkaline environment neutralizes lactic acid from osteoclasts, avoiding dissolution of the dentin mineral components. Calcium ions can induce expressions of type I collagen, osteopontin, osteocalcin, and alkaline phosphatase enzyme in osteoblasts and mineralization through the phosphorylation of p38 mitogen-activated protein kinase and c-Jun N-terminal kinase. Alkaline phosphatase liberates inorganic phosphatase from phosphate esters. It can separate phosphoric esters, releasing phosphate ions that react with bloodstream calcium ions to form calcium phosphate of hydroxyapatite [17].

Bone morphogenetic protein- (BMP-) 2 is a growth factor that is expressed in presence of calcium hydroxide. BMP-2 aids the regeneration of bone, cementum, and periodontal tissue. It may act as a mitogen for undifferentiated mesenchymal cells and osteoblast precursors, inducing osteoblast phenotype expression, and as a chemoattractant for mesenchymal cells and monocytes. Additionally, BMP-2 may bind to extracellular matrix type IV collagen [17]. Calcium hydroxide also creates a necrotic zone by rupturing glycoproteins in the intercellular substance, resulting in protein denaturation and granulation tissue [18].

The paste vehicle may also play an important role in treatment. The vehicle determines the velocity of ionic dissociation. It allows the paste to be solubilized and resorbed by the periapical tissues and from within the canal. The lower the viscosity, the higher the ionic dissociation [3]. The viscosity of propylene glycol minimizes the dispersion of calcium hydroxide into the tissues and maintains the paste in the desired area for longer periods of time. This vehicle also presents biocompatibility and antibacterial activity [19].

However, as mentioned above, calcium hydroxide has some drawbacks. The highly necrotic zone in the periapical bone can be managed by careful material placement and radiographic control, avoiding overfilling of the canal. Tooth prognostic compromise due to the lack of a definitive coronal restoration can be avoided by using suitable operative procedures and adhesive restorations. The risk of cervical root fracture due to long-term dressing may be decreased by using a mutually protected occlusal scheme and patient instruction or by limiting the material placement below the cervical limit.

Long-term exposure of dentine to hydroxyl ions reduces the flexural strength and fracture resistance [19]. Ion diffusion depends on the regional diameter of dentin tubules and the presence of a smear layer [20]. In the present case, the conventional application of 17% EDTA for 3 min under instrumental agitation was not used to remove the smear layer. The immature tooth did not need substantial instrumentation of the canal walls. It was assumed that the quantity of smear layer produced would not prevent the antimicrobial effect of sodium hypochlorite but probably would avoid the excessive diffusion of ions, perhaps decreasing the risk of fracture.

MTA is used as an alternative to calcium hydroxide. Its main advantage is lower treatment time, with minimal delay before placing a definitive restoration, decreasing the prolonged highly alkaline effect of calcium hydroxide and coronal leakage [1]. However, there is insufficient evidence about its healing superiority [2]. Moreover, MTA shows some disadvantages, such as difficulty in handling, extended setting time, irreversible application, and higher cost [18]. The present case was completed in 1999, when the evidence about apexification using MTA was still incipient.

The final radiograph (Figure 7), corresponding to 14 and half years after treatment, shows a slight radiolucent image around the periapical area. However, this form is probably a visual effect from trabecular spaces, a space corresponding to periodontal ligament or a particular structural sinuosity of apex, since the apexification can be visualized in Figure 9 after 16 years and two months.

## 4. Conclusion

The findings in this case report suggest that calcium hydroxide provides a viable alternative to achieve root end closure in an immature tooth. Despite the limitations of this material, the described technique permitted satisfactory apexification treatment in the long-term.

## References

[1] Moore A., Howley M. F., O'Connell A. C. (2011). Treatment of open apex teeth using two types of white mineral trioxide aggregate after initial dressing with calcium hydroxide in children. *Dental Traumatology*.

[2] Bakland L. K., Andreasen J. O. (2012). Will mineral trioxide aggregate replace calcium hydroxide in treating pulpal and periodontal healing complications subsequent to dental trauma? A review. *Dental Traumatology*.

[3] Mohammadi Z., Dummer P. M. H. (2011). Properties and applications of calcium hydroxide in endodontics and dental traumatology. *International Endodontic Journal*.

[4] Yassen G. H., Chin J., Mohammedsharif A. G., Alsoufy S. S., Othman S. S., Eckert G. (2012). The effect of frequency of calcium hydroxide dressing change and various pre- and inter-operative factors on the endodontic treatment of traumatized immature permanent incisors. *Dental Traumatology*.

[5] Finucane D., Kinirons M. J. (1999). Non-vital immature permanent incisors: factors that may influence treatment outcome. *Endodontics and Dental Traumatology*.

[6] Strom T. A., Arora A., Osborn B., Karim N., Komabayashi T., Liu X. (2012). Endodontic release system for apexification with calcium hydroxide microspheres. *Journal of Dental Research*.

[7] DiAngelis A. J., Andreasen J. O., Ebeleseder K. A. (2012). International association of dental traumatology guidelines for the management of traumatic dental injuries: 1. Fractures and luxations of permanent teeth. *Dental Traumatology*.

[8] Frank A. L. (1966). Therapy for the divergent pulpless tooth by continued apical formation. *The Journal of the American Dental Association*.

[9] Ballesio I., Marchetti E., Mummolo S., Marzo G. (2006). Radiographic appearance of apical closure in apexification: follow-up after 7–13 years. *European Journal of Paediatric Dentistry*.

[10] Soares A. D. J., Nagata J. Y., Casarin R. C. V. (2012). Apexification with a new intra-canal medicament: a multidisciplinary case report. *Iranian Endodontic Journal*.

[11] Tarján I., Gyulai G. S., Gábris K. (2004). Literature survey of apexification in connection with three cases. *Fogorvosi Szemle*.

[12] Thäter M., Maréchaux S. C. (1988). Induced root apexification following traumatic injuries of the pulp in children: follow-up study. *ASDC Journal of Dentistry for Children*.

[13] Chawla H. S. (1991). Apexification: follow-up after 6–12 years. *Journal of Indian Society of Pedodontics & Preventive Dentistry*.

[14] Gaitonde P., Bishop K. (2007). Apexification with mineral trioxide aggregate: an overview of the material and technique. *The European journal of prosthodontics and restorative dentistry*.

[15] Ohara P. K., Torabinejad M. (1992). Apical closure of an immature root subsequent to apical curettage. *Endodontics & Dental Traumatology*.

[16] Silva L. A. B., Nelson-Filho P., Leonardo M. R., Rossi M. A., Pansani C. A. (2002). Effect of calcium hydroxide on bacterial endotoxin in vivo. *Journal of Endodontics*.

[17] Ham K. A., Witherspoon D. E., Gutmann J. L., Ravindranath S., Gait T. C., Opperman L. A. (2005). Preliminary evaluation of BMP-2 expression and histological characteristics during apexification with calcium hydroxide and mineral trioxide aggregate. *Journal of Endodontics*.

[18] Estrela C., Holland R. (2003). Calcium hydroxide: study based on scientific evidences. *Journal of Applied Oral Science*.

[19] Fava L. R. G., Saunders W. P. (1999). Calcium hydroxide pastes: classification and clinical indications. *International Endodontic Journal*.

[20] Violich D. R., Chandler N. P. (2010). The smear layer in endodontics—a review. *International Endodontic Journal*.

